# The Impact of Epistemic Curiosity on Traffic Risky Behavior: The Mediating Role of Conformity

**DOI:** 10.1002/pchj.70024

**Published:** 2025-06-19

**Authors:** YiMeng Cui, DongYang Wang, XiaoCai Gao

**Affiliations:** ^1^ Institute of Applied Psychology, School of Public Management Northwest University Shaanxi China

**Keywords:** conformity, deprivation curiosity, epistemic curiosity, interest curiosity, traffic risky behavior

## Abstract

Different types of epistemic curiosities are associated with opposite attitudes toward risky behavior. However, few studies have taken environmental factors into account. We do not know the specific performance of different curiosities regarding traffic risky behavior (TRB) after introducing public attitudes. Epistemic curiosity is the desire for new knowledge or information. There are two types: interest curiosity and deprivation curiosity. Based on the uncertainty‐identity theory and the interest/deprivation model of curiosity, we explored the impact of epistemic curiosity on TRB and the mediating role of conformity. Study 1 employed a cross‐sectional design with mediation effect tests. Study 2 employed two substudies, further exploring the specific performance of different levels of interest/deprivation curiosity through a 2 × 2 mixed design and elaborating on the causal relationships between the variables. Study 1 revealed a positive correlation between interest curiosity and TRB, but no such correlation was found between deprivation curiosity and TRB. Mediation test results showed that conformity fully mediated the relationship between deprivation curiosity and TRB, while it could not explain the relationship between interest curiosity and TRB. Study 2 results showed that people with higher levels of deprivation curiosity could be influenced by public attitudes to change their attitudes toward TRB. Our findings provide empirical evidence for distinguishing between different types of epistemic curiosity, as well as a new explanatory mechanism for the emergence of TRBs.

## 1 Introduction

Curiosity is defined as an internally driven exploration aimed at acquiring a better understanding or knowledge (Comunian 1994; Loewenstein 1994). Berlyne (1962) first divided curiosity into four categories: diverse curiosity, specific curiosity, perceptual curiosity, and epistemic curiosity (Li et al. 2023, 1). Epistemic curiosity is an exploratory motivation driven by lacking knowledge‐based information, like knowledge and theories, which is unique to humans (Berlyne 1954; Litman and Jimerson 2004). Litman (2008) proposed two types of epistemic curiosity: interest curiosity and deprivation curiosity. Research indicated that they showed a paradoxical relationship with risky behaviors. Interest curiosity was related to active exploration and less concerned with the risks associated with the behavior; however, deprivation curiosity was more concerned with the risks of things and more cautious when confronted with exploratory behaviors (Lauriola et al. 2015).

Additionally, the study of the relationship between epistemic curiosity and risky behavior cannot ignore the effect of societal influence. In the two types of epistemic curiosity, deprivation curiosity is closely related to conformity. Deprivation curiosity is more likely to be influenced by popular attitudes and develop a tendency to conformity, but interest curiosity is not. Specifically, being in a state of deprivation, individuals would seek out negative information in order to comply with social norms (Niehoff and Oosterwijk 2020). Conformity also affects people's attitudes toward risky behaviors, causing them to ignore obvious potential risks. For example, when under the influence of peer pressure, people might disregard their own safety and engage in risky behaviors such as traffic violations, smoking, drinking, or even drug use (Hess and de Almeida 2019; Jongenelis et al. 2019; Pierce et al. 2005; Rulison et al. 2016).

Summarily, the relationship between different types of epistemic curiosity and risky behavior is quite different. Interest curiosity will be more inclined to engage in risk‐taking, while deprivation curiosity will reject it. However, when we include conformity into the process, different types of epistemic curiosity will show a different relationship with risky behaviors than they do before. That is, people with deprivation curiosity would engage in risky behaviors due to the tendency to conform. Up to now, however, few studies have explored the explanatory mechanisms and specific manifestations behind this phenomenon. Our research aims to explore the relationship between epistemic curiosity and traffic risky behavior (TRB) and the mediating role of conformity in this relationship.

### 1.1 Curiosity and Risky Behavior

Being curious means more exploratory behaviors toward things we cannot understand or want to learn more about (Horn et al. 2024; Sekiguchi 2023; Whitecross and Smithson 2023a). These exploratories have many benefits. People with curiosity have longer lifespans (Swan and Carmelli 1996) and higher life satisfaction (Jovanovic and Brdaric 2012). Regarding problem‐solving ability and performance, curious people exhibit greater creativity, higher exam scores, and better job performance and management skills (Anderson et al. 2020; Hardy III et al. 2017; Hassan et al. 2015; Huck et al. 2020; Kang et al. 2009; Ma 2023; Tang and Salmela‐Aro 2021).

Even researchers claimed that high curiosity fosters a wealth of psychological benefits. That does not mean we can ignore the danger that curiosity brings. People with curiosity tend to engage in many risky behaviors, which might lead to danger. They would also believe the false information (Zedelius et al. 2022) and choose to watch the information that is already known as dangerous (Oosterwijk 2017). Hsee and Ruan (2016) employed a series of experiments to provide systematic and in‐depth research on the dark side of curiosity. During the experiments, participants were provided with a variety of disgusting stimuli. The final results showed that participants chose to explore the information even though they were told in advance that touching the materials or choosing to look at the stimuli would cause physical discomfort.

### 1.2 Epistemic Curiosity and Traffic Risky Behavior

Epistemic curiosity is specifically generated in response to the information gap (Berlyne 1962; Litman and Spielberger 2003; Whitecross and Smithson 2023a). There are two types of epistemic curiosity: interest curiosity and deprivation curiosity. Interest curiosity refers to curiosity about things arising from an individual's interests (Litman and Spielberger 2003). It can lead to happy exploration. In contrast, deprivation curiosity refers to curiosity about things arising from the relative sense of deprivation. It can lead to anxious persistent exploration and seeking behaviors (Lauriola et al. 2015; Litman 2010; Litman and Jimerson 2004). Litman (2010) further proposed the interest/deprivation model of curiosity to explain the differences between the two types of epistemic curiosity. Both interest and deprivation curiosity have a high level of liking for things, but interest curiosity has a lower level of wanting for things than deprivation curiosity. Moreover, interest curiosity has tolerance for ambiguity, but deprivation curiosity cannot tolerate ambiguity in things.

Numerous research also provided evidence for the interest/deprivation model of curiosity. For psychological aspects, interest curiosity was significantly related to well‐being, especially academic well‐being, whereas deprivation curiosity was not (Li et al. 2023). Moreover, interest curiosity was positively correlated with positive outcome expectations and more positive emotions, whereas deprivation curiosity was positively correlated with negative outcome expectations and more negative emotions (Whitecross and Smithson 2023a). For behavioral performance, participants engaged in more unnecessary behaviors that were unrelated to the goal when deprivation curiosity was aroused (Shen et al. 2020). When confronted with false information, people with high deprivation curiosity scores are more likely to lack intellectual humility and are more likely to believe that (Zedelius et al. 2022).

TRB is a type of risky behavior that poses a threat to the individual's health, primarily when they are a pedestrian or driver (Vickers Jr. et al. 1990). TRBs are widespread and can cause major accidents and irreversible damage (Zhang et al. 2023; Nicolls et al. 2024; Vardaki and Yannis 2013). Research showed that individual psychological factors can predict their TRB (Kochetova 2022; Lemarié et al. 2019). Risk perception is an important predictive indicator of TRB (Ulleberg and Rundmo 2003; Carter et al. 2014). Drivers who perceive high traffic risks were shown to exhibit less risky behavior during driving. When crossing the road, pedestrians with higher perceived risks were also shown to exhibit safer traffic behaviors (Rosenbloom et al. 2016).

The interest/deprivation model of curiosity proposed that interest curiosity would tend to take risks, whereas deprivation curiosity was not related to risky behaviors (Lauriola et al. 2015). Lauriola et al.'s (2015) study indicated that deprivation curiosity was correlated with risk perception, whereas interest curiosity was not. When considering some risky behaviors, people with deprivation curiosity tend to consider the risk behind the behavior, whereas people with interest curiosity do not. When considering risky cues, drivers would adjust their driving strategies and avoid risky behaviors. However, those with low‐risk perceptions would engage in TRBs such as not wearing a seatbelt, drunk driving, and speeding (Ryb et al. 2006). Therefore, we hypothesize a paradoxical relationship between epistemic curiosity and traffic risk behaviors. The hypotheses are as follows:

Hypothesis 1a *Interest curiosity is positively correlated with traffic risky behavior*.

Hypothesis 1b *Deprivation curiosity is not positively correlated with traffic risky behavior*.

### 1.3 Epistemic Curiosity and Conformity

Conformity is a kind of social phenomenon that encourages individuals to change their personal opinions and behaviors to agree with the majority (Asch 1951; Hertz and Wiese 2016; Larsen 1974). Uncertainty is an important cause of conformity (Kruglanski and Webster 1991; Smith et al. 2007; Kruglanski et al. 2006). Uncertainty‐identity theory proposes that conformity is a useful strategy to reduce uncertainty. When people are in a state of high uncertainty, they tend to identify with the group to reduce uncertainty and satisfy a sense of social belonging (Yang et al. 2021; Hogg 2007; Hogg et al. 2010).

Based on Litman's (2010) interest/deprivation model of curiosity, epistemic curiosity may have a paradoxical relationship with conformity. Compared to interest curiosity, people with deprivation curiosity have a lower tolerance for uncertainty (Whitecross and Smithson 2023b). They are more susceptible to public attitudes. Research also showed that when facing uncertainty, those with high relative deprivation tend to listen to the public to alleviate anxiety caused by uncertainty (Smith et al. 2007). Even when aware of high risks, they are also seeking the corresponding information (Mechera‐Ostrovsky et al. 2023). In summary, the hypotheses are as follows:

Hypothesis 2a *Interest curiosity is not positively correlated with conformity*.

Hypothesis 2b *Deprivation curiosity is positively correlated with conformity*.

### 1.4 The Medicating Role of Conformity

Uncertainty‐identity theory proposes that people easily conform when they are in uncertain situations. Even when they have been informed that the behavior is harmful, people are willing to follow groups to maintain their sense of social belonging (Hogg et al. 2011; Ouyang et al. 2024). This theory proved a theoretical rationale for the relationship between conformity and TRB. Conformity is an important factor affecting pedestrians' intention to violate traffic rules and accident proneness (Tang et al. 2021). Previous literature indicated that the tendency to follow the majority could influence people's traffic performance and that individuals with a higher conformity tendency were more likely to follow the influence of those around them in their traffic behavior (Zhou et al. 2009), especially among adolescents (Zhou and Horrey 2010). Pedestrians with their companions were more likely to cross the road illegally compared to themselves alone (Zhang et al. 2023). Thus, we believe that TRB is the consequence of people with deprivation curiosity changing themselves by following public attitudes. The hypotheses are as follows:

Hypothesis 3a *Conformity does not play a mediating role between interest curiosity and traffic risky behavior*.

Hypothesis 3b *Conformity plays a mediating role between deprivation curiosity and traffic risky behavior*.

Currently, most studies on the differences between different types of epistemic curiosity use questionnaires, with few relevant experimental studies. This study distinguishes between two types of epistemic curiosity. After considering conformity, we explored the attitude change of two types of epistemic curiosity toward TRB. We used the experimental design to test the proposed hypothesis, providing empirical evidence for distinguishing different categories of epistemic curiosity.

## 2 Overview of the Two Studies

We used two studies to test our hypothesis. Study 1 used the questionnaire method. First, we used descriptive statistics and correlation analysis of the results to expose the correlations between different types of epistemic curiosity, conformity, and traffic risk behaviors. Then we used the mediation effects test to examine the mediating role of conformity between epistemic curiosity and TRB.

Study 2 was divided into two substudies based on the different types of epistemic curiosity. We wanted to separately explore the specific attitude change of interest (deprivation) curiosity toward the TRB in conformity scenarios. When considering the performances of participants with deprivation curiosity, we took control by screening them for consistent levels of interest curiosity. Then, we investigated the attitudes of participants with different levels of deprivation curiosity toward TRBs in the conformity environment. When considering the performance of participants with interest curiosity, their deprivation curiosity was controlled at the same level.

## 3 Study 1

### 3.1 Methods

#### 3.1.1 Participants

We recruited 305 (50.2% female, *n* = 153; 49.8% male, *n* = 152) nonpsychology college students whose native language is Chinese. Participants ranged in age from 18 to 26 (*M* = 21, SD = 1.65). Participants included 256 (83.9%) undergraduate students and 49 (16.1%) master's students. Informed consent was obtained before the experiment, and each participant received a gift worth 5 RMB. The content of this study has been approved by the ethics committee of Northwest University.

### 3.2 Measures

#### 3.2.1 Epistemic Curiosity

Epistemic curiosity was assessed by the epistemic curiosity scale, which is composed of a 5‐item interest curiosity subscale (e.g.,“liking to explore new ideas”), and a 5‐item deprivation curiosity subscale (e.g.,“I need to think for a long time to solve problems”) (Litman 2008). Each item is rated on a 4‐point Likert scale from 1 (*almost never*) to 4 (*almost always*). Good reliability and validity were supported in the Chinese university samples (Tang and Salmela‐Aro 2021). The alpha coefficient of the epistemic curiosity scale in this study was 0.86. The alpha coefficient of the interest curiosity scale was 0.77, and the deprivation curiosity scale was 0.73.

#### 3.2.2 TRB

TRB was assessed by selecting 7‐item from the risk dimension for this study (e.g., “I run a red light when crossing the road”) (Vickers et al. 1990). Each item is rated on a 5‐point Likert scale from 1 (*almost never*) to 5 (*almost always*). Good reliability and validity were supported in the Chinese university samples (Mo and Winnie 2010). The alpha coefficient of the scale in this study was 0.78.

#### 3.2.3 Conformity

Conformity was assessed by social information comparison scale, which is a 13‐item scale (e.g., “I proactively avoid wearing inappropriate clothes”) (Lennox and Wolfe 1984). Each item is rated on a 6‐point scale from 0 (*nonconformance*) to 5 (*very conformance*). Good reliability and validity were supported in the Chinese university samples (Wu and Chang 2012). The alpha coefficient of the scale in this study was 0.83.

### 3.3 Analyses

We used IBM SPSS Statistics 26.0 to calculate the descriptive statistics and correlations of tested variables. An intermediate effect test was conducted using the PROCESS3.3 (Model 4) program with 5000 bootstrapped samples (Hayes 2013).

### 3.4 Results

#### 3.4.1 Description Statistics

Table 1 shows the descriptive statistics of the scales and bivariate correlations between the scale scores included in the present study. The correlation between interest curiosity and deprivation curiosity was *r* = 0.74 (*p* < 0.01), which has a certain degree of collinearity. To evaluate the particular relationship between each epistemic curiosity and other variables, we computed partial correlations referring to previous studies (Lauriola 2015).

**TABLE 1:** Mean, standard deviation, and correlation among study variables in Study 1 (*N* = 305).

Variables	*M*	SD	1	2	3	4	5	6	7
1.Gender	0.50	0.50	—						
2.Age	21.11	1.65	−0.02	—					
3.Education	0.17	0.41	0.12*	0.44**	—				
4.Interest curiosity	15.06	2.73	−0.15**	0.05	0.01	—			
5.Deprivation curiosity	15.00	2.73	−0.09	0.11	0.02	0.74**	—		
6.TRB	10.96	5.84	−0.23**	0.08	0.121*	0.20**	0.15*	—	
7.Conformity	36.56	9.63	−0.10	0.09	−0.01	0.34**	0.34**	0.18**	—

*Note*: Gender conversion to dummy variable (1 = female; 0 = male); Education conversion to dummy variables (0 = undergraduate; 1 = master).

Abbreviation: TRB, traffic risky behavior.

* *p* < 0.050.

** *p* < 0.01.

Each variable was tested separately for partial correlation with interest, curiosity, and deprivation curiosity. The zero‐order and partial correlations are shown in Table 2.

**TABLE 2:** Zero‐order and partial correlations between the interest and deprivation curiosity scales and conformity and traffic risky behavior in Study 1 (*N* = 305).

	Zero‐order		Partial *r*	
	IC	DC	IC	DC
Interest curiosity				
Deprivation curiosity				
Conformity	0.34**	0.34**	0.14*	0.14*
TRB	0.20**	0.15*	0.14*	−0.004

Abbreviations: DC, deprivation curiosity; IC, interest curiosity; TRB, traffic risky behavior.

* *p* < 0.05.

** *p* < 0.01.

After controlling the score of deprivation curiosity, interest curiosity and TRB had a weak positive correlation (*r =* 0.14, *p* < 0.05); H1a was verified. After controlling the score of interest curiosity, deprivation curiosity and TRB had no correlation (*r = −*0.004, *p* > 0.05); H1b was verified. After controlling the score of deprivation curiosity, interest curiosity and conformity had a weak positive correlation (*r =* 0.14, *p* < 0.05); H2a was not verified. After controlling the score of interest curiosity, deprivation curiosity and conformity had a weak positive correlation (*r =* 0.14, *p* < 0.05); H2b was verified.

#### 3.4.2 Analysis of Mediating Effect

Testing the mediating effect between variables. Gender, age, and education were used as control variables (González‐Iglesias et al. 2012; Qian et al. 2020). The result is shown in Table 3.

**TABLE 3:** Comparison of mediating model test of conformity in interest and deprivation curiosity and traffic risky behavior in Study 1.

Paths	B	SE	95 bce% CI	
Interest curiosity				
Total effects	0.17	0.06	0.06	0.27
Direct effects	0.13	0.06	0.01	0.24
Indirect effects	0.04	0.02	−0.003	0.08
Deprivation curiosity				
Total effects	0.12	0.06	0.01	0.23
Direct effects	0.08	0.06	−0.04	0.19
Indirect effects	0.04	0.02	0.003	0.09

**FIGURE 1:**
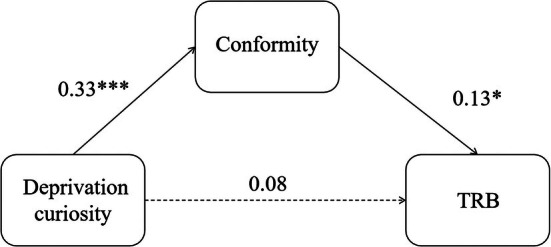
Research the mediating effect model diagram of the conformity, deprivation curiosity, and TRB in Study 1. TRB, traffic risky behavior. *p < 0.05, ***p < 0.001.

Mediating effect test result shows in Table 3. In the model of conformity with deprivation curiosity and TRB, the direct effect was not significant (*p =* 0.176 > 0.05; 95% CI [−0.04, 0.19]), the indirect effect (95% CI [0.003, 0.09]) and total effect were significant (*p =* 0.029 < 0.05; 95% CI [0.01, 0.23]). In the models of conformity, interest curiosity, and TRB, the indirect effect was not significant (95% CI [−0.003, 0.08]); this model mediating effect was not established. The mediation model diagram of deprivation curiosity, conformity, and TRB models was shown in Figure 1.

### 3.5 Discussion

Results of partial correlation in descriptive statistics showed a weak correlation between interest curiosity and TRB and a moderate correlation between interest curiosity and conformity. There was no correlation between deprivation curiosity and TRB, but a significant correlation with conformity. H1a, H1b and H2b were valid, but H2a was not. Compared with previous studies, our results were consistent with Litman's interest/deprivation model of curiosity. Individuals with high interest curiosity show a higher willingness to take risks, whereas individuals with high deprivation curiosity worry about potential risks and are reluctant to engage in risky behavior (Lauriola et al. 2015; Litman 2010). After analysis, the relationship between deprivation curiosity and conformity in this study was consistent with the previous study, where individuals in a state of deprivation were more inclined to follow the opinions of the majority (Smith et al. 2007). However, the relationship between interest curiosity and conformity was different from previous studies (Whitecross and Smithson 2023b): individuals showed the same tendency to conformity even when in the state of interest.

In the mediating effect test results, the indirect effect of interest curiosity and TRB was not significant, while the indirect effect of deprivation curiosity and TRB was significant. The overall effect was significantly consistent with H3b, indicating that conformity mediated the relationship between deprivation curiosity and TRB. Results showed that conformity fully mediated the relationship between deprivation curiosity and traffic risk behavior, but could not explain the relationship between interest curiosity and TRB.

## 4 STUDY 2

Compared to experiments, questionnaires cannot test the causal relationship between variables. Therefore, we set up the conformity situations experimentally in Study 2, wanting to explore the causal relationship between high and low levels of interest/deprivation curiosity and TRBs. Specifically, we used two substudies to examine this study. When examining the role of deprivation curiosity, we used a prequestionnaire to select participants with equal interest curiosity scores. They were divided into high and low groups according to their deprivation curiosity scores and put into a conformity situation to observe the change of attitude toward TRBs. When examining the role of interest curiosity, we screened participants with the same deprivation curiosity score.

### 4.1 Methods

#### 4.1.1 Participants

In the early stage, we tracked 455 college students. Overall average interest curiosity score (*M* = 14.6, SD = 2.6) and deprivation curiosity (*M* = 14.3, SD = 2.5) score were obtained by filling out the epistemic curiosity scale (same as Study 1). Participants who scored outside of one standard deviation of the average (equal to or higher than 17, equal to or less than 12) were selected for the experiment of Study 2. There are 121 participants in total (62.8% female, *n* = 76; 37.2% male, *n* = 45), with an average age of 20.82 (SD = 1.68) and an age range of 18–26. Participants included 105 (86.8%) undergraduate students and 16 (13.2%) master's students. In the high deprivation curiosity score group, there were 28 (93.3%) undergraduate and two (6.7%) master's students; in the low deprivation curiosity score group, there were 24 (80%) undergraduate and six (20%) master's students. In the high interest curiosity score group, there were 29 (93.5%) undergraduate students and two (6.5%) master's students; in the low interest curiosity score group, there were 24 (80%) undergraduate students and six (20%) master's students. Informed consent was obtained on a computer screen before the experiment, and each participant received a gift worth 5 RMB. The content of this study has been approved by the Northwest University ethics committee.

#### 4.1.2 Material

Images and descriptions used in the experiment adopt the content of the TRB in Study 1. We changed the items of the traffic risk behavior scale in Study 1 to images and brief descriptions and described each item with two sets of images and text and set up different public attitude displays for each of them. We use mosaic to blur the image to simulate the situation where the individual is half‐aware of TRBs.

#### 4.1.3 Design and Procedure

Study 2 contained two substudies that explored the different performances of people with high or low interest/deprivation curiosity scores. Our experimental program was adapted from the classic conformity paradigm (Huang et al. 2014), which was compiled by Eprime 3.0. The experiment for each substudy was a 2 × 2 mixed experimental design. Public attitude was a within‐subjects variable with two levels: same and opposite; curiosity level was a between‐subjects variable with two levels: high and low.

Curiosity level manipulations were as follows: in the early stage, we used a large‐scale subject questionnaire to derive the mean scores and standard deviations of college students on interest curiosity and deprivation curiosity. When considering the performance of deprivation curiosity, participants who scored no significant difference in interest curiosity were selected by matching. The high‐level group scores more than the mean by one standard deviation, and the low‐level group scores less than the mean by one standard deviation. When considering interest curiosity, the steps were consistent with the previous ones. Public attitude manipulation was as follows: we provided two opposed attitudes for each traffic risk behavior image, ensuring that participants did not see two attitudes toward the same behavior consecutively by pairing the materials as well as randomizing the display. We measured the number of changes in the attitudes of participants.

Before the experiment began, we informed participants that we were conducting a health behavioral intention survey. Specifically, we would give them some blurry images and a short description of each image, all of which were about TRBs. They had two options: “watch” and “skip”; “watch” means interested in the behavior and would like to try it if they get the chance, and “skip” means not interested and will not try it. We collected the opinions of 200 people and marked them with the red box in the experiment. Before the formal phase of the experiment began, participants were asked again if they understood and remembered the meaning of the two options.

The experimental procedure had two stages. First, a blurred image and a description of its content were presented on the screen with two options: “watch” and “skip.” Participants were asked to choose. After they had made their choice, it was marked with a green box. Then, one of the two options was marked with a red box, informing them that this option was the public attitude. Later, participants were given a screen with “Please prepare and proceed to the next stage.” In the second stage, blurred images and descriptions were shown again. Participants were asked to choose again. During the experiment, participants should stare at the gaze to maintain attention.

### 4.2 Analyses

Before analysis, we log‐transformed the data to prevent it from being too scattered. Using an independent sample *t* test to test whether there was a significant difference in the level of curiosity score, levels of demographic variables, and other dimensions except for the target variable (González‐Iglesias et al. 2012; Qian et al. 2020). The data was analyzed using repeated measures of variance analysis (ANOVA). IBM SPSS 26.0 was used for the above experimental data analysis.

### 4.3 Results

#### 4.3.1 Deprivation Curiosity

Conducted independent sample *t* tests to analyze the effects of gender, age, education, and interest curiosity score among experimental participants. As shown in Table 4, there was no significant difference in the above variables between the two groups of participants (Qian et al. 2020).

**TABLE 4:** Comparison of other demographic characteristics between two groups of participants with high and low deprivation curiosity scores in Study 2.

	High‐scoring group (*n* = 30)		Low‐scoring group (*n* = 30)		*t*
	*M*	SD	*M*	SD	
Gender	0.60	0.50	0.70	0.47	−0.80
Age	20.83	1.09	21.23	1.76	−1.06
Education	0.07	0.25	0.20	0.41	−1.52
Interest curiosity	14.93	1.05	14.40	1.16	1.87

*Note*: Gender conversion to dummy variable (1 = female; 0 = male); Education conversion to dummy variables (0 = undergraduate; 1 = master).

**FIGURE 2:**
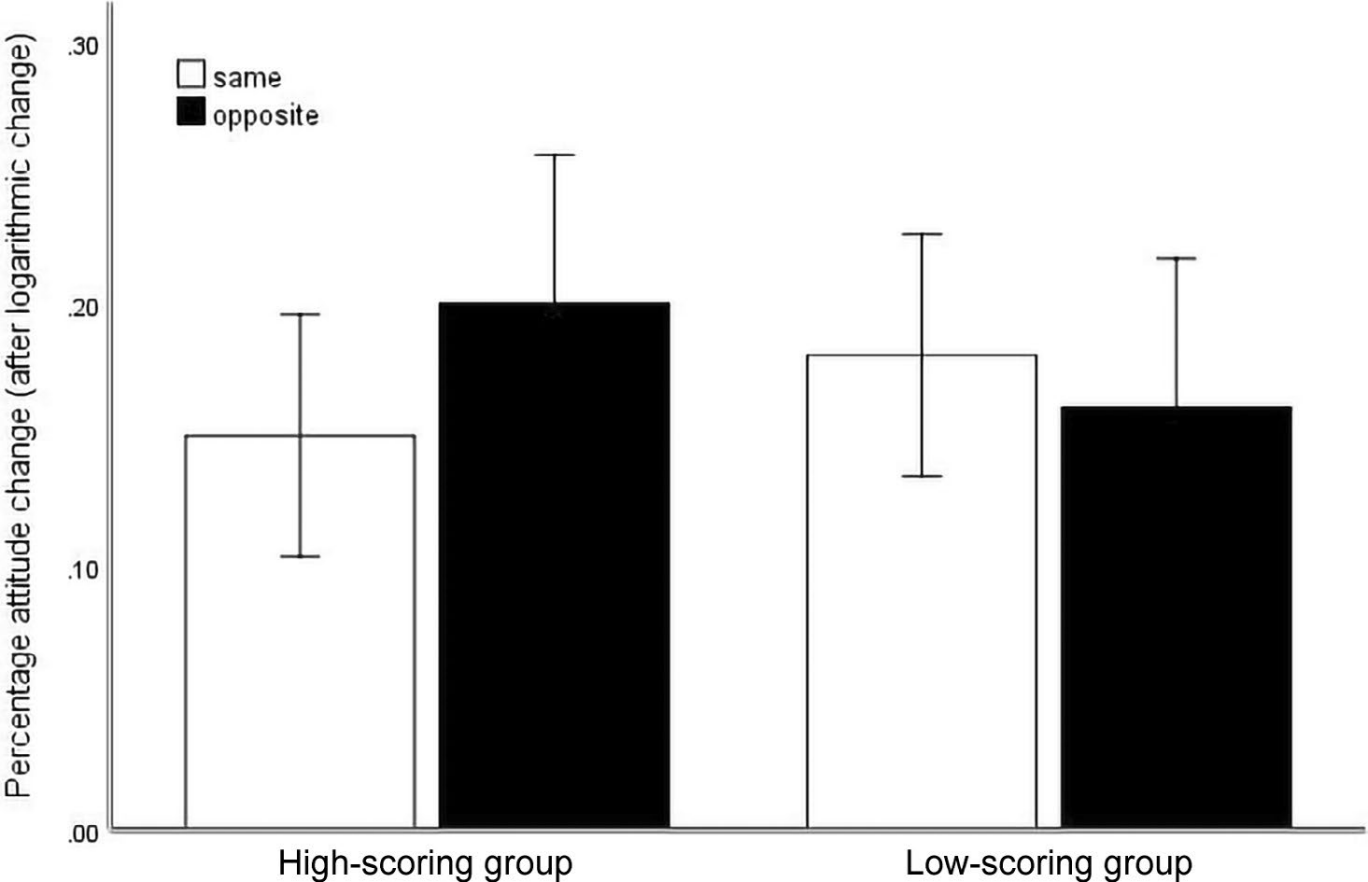
Interaction between deprivation curiosity and public attitude in Study 2. *Note*: Dividing score group into low and high groups; Dividing public attitude into same and opposite groups; Result is within 95% confidence interval; Y‐axis represents the logarithmic transformation result of the percentage change in participants' options.

Analyses of individuals' attitude change toward TRB under deprivation curiosity and different public attitudes are shown in Figure 2. The interaction between deprivation curiosity and the consistency of public attitudes was moderately significant (*F*(1, 1) = 4.44, *p =* 0.039 < 0.05, Cohen's *f* = 0.28). For the simple effect analysis, it was found that under the conditions that public attitude was opposite to their own, individuals with high scores in deprivation curiosity had more significant attitude changes (*M* = 0.20, SD = 0.17, 95% CI [0.003, 0.097]) than under the same conditions (*M* = 0.15, SD = 0.13). But there was no significant difference in the level of performance of low deprivation curiosity between consistent (*M* = 0.18, SD = 0.12, 95% CI [−0.027, 0.067]) and inconsistent (*M* = 0.16, SD = 0.14) situations.

#### 4.3.2 Interest Curiosity

We conducted independent sample *t* tests to analyze the effects of gender, age, education, and deprivation curiosity score among experimental participants. As shown in Table 5, there was no significant difference in the above variables between the two groups of participants (Qian et al. 2020).

**TABLE 5:** Comparison of other demographic characteristics between two groups of participants with high and low interest curiosity scores in Study 2.

	High‐scoring group (*n* = 31)		Low‐scoring group (*n* = 30)		*t*
	*M*	SD	*M*	SD	
Gender	0.52	0.51	0.70	0.47	−1.47
Age	20.61	1.67	20.60	2.06	0.03
Education	0.06	0.25	0.20	0.41	−1.57
Deprivation curiosity	14.29	1.04	13.83	0.79	1.94

*Note*: Gender conversion to dummy variable (1 = female; 0 = male); Education conversion to dummy variables (0 = undergraduate; 1 = master).

Results of repeated measures ANOVA for the high and low interest curiosity score groups showed that the main effect of conformity was moderately significant (*F*(1, 1) = 4.67, *p =* 0.035 < 0.05, Cohen's *f* = 0.28), which indicated that our manipulation of the experiment was effective. The interaction between interest curiosity and conformity was not significant (*F*(1, 1) = 2.13, *p =* 0.150 > 0.05).

### 4.4 Discussion

In Study 2, we focused on the changing attitudes of interest curiosity and deprivation curiosity toward TRB in conformity scenarios, aiming to further explore the specific performances of different types of epistemic curiosity.

When there was no significant difference in interest curiosity scores, individuals with higher deprivation curiosity scores would be more easily influenced by public attitudes when faced with behaviors that they knew were risky. This was consistent with Tang et al.'s (2021) study. Individuals in a deprivation state tend to be influenced by group pressure when making decisions, even disregarding the potential risks involved. Among participants with no differences in deprivation curiosity scores, neither individuals with high nor low interest curiosity scores showed altered attitudes toward traffic risk behaviors in response to public attitudes.

## 5 General Discussion

Based on the uncertainty‐identity theory and the interest/deprivation model of curiosity, our research explored the relationship between epistemic curiosity and traffic risk behavior. We also revealed mechanisms among deprivation curiosity, traffic risk behavior, and conformity. Our central idea is that different types of epistemic curiosity display different attitudes when facing risky behaviors. Conformity could provide a partial explanation for this. Specifically, when only considering the relationship between epistemic curiosity and traffic risk behavior, interest curiosity leads to TRB, whereas deprivation curiosity tends to avoid it. However, the situation will be different when putting them into the conforming environment. In the conformity scenario, interest curiosity would not change attitudes toward TRBs, whereas deprivation curiosity would instead be disturbed by public attitudes. Our research has both theoretical and practical implications.

### 5.1 Theoretical Implications

From the theoretical viewpoint, first, our findings align with Litman's (2010) interest/deprivation model of curiosity. Interested curiosity was positively related to risky behavior, whereas deprivation curiosity was not. Our findings provided useful evidence for the literature on the differences between these two types of epistemic curiosity (Litman and Spielberger 2003; Lauriola et al. 2015; Whitecross and Smithson 2023a, 2023b). Meanwhile, the introduction of conformity scenes could improve the applicability and prediction accuracy of the model. Furthermore, we build on existing theories to provide a new explanatory framework for the emergence of curiosity about risky behaviors. That is, personal risky behavior is the result of different types of epistemic curiosity influenced by the public. Our findings provide evidence for this framework: People with high deprivation curiosity could be influenced by public attitudes, thereby changing attitudes about knowingly risky behaviors. Finally, our study integrated conformity and risky behaviors to provide a richer perspective on the field of curiosity research. That is, whether curiosity‐driven information seeking attitudes exhibit the opposite when considering the influence of the environment.

### 5.2 Practical Implications

From the practical viewpoint, first, our finding provided a reference significance for reducing individual participation in risky behaviors. In real life, there are risky behaviors that are well known, such as drinking, smoking, and traffic violations (Hess and de Almeida 2019; Jongenelis et al. 2019; Pierce et al. 2005). Our finding indicated that our attitude toward these risky behaviors might be influenced by public attitudes or peers. Curiosity arising from the deprivation sense is more susceptible to this influence. From our findings, we could intervene in personal deprivation when considering future risky behavioral disturbances, reducing or even eliminating personal risky behaviors by reducing an individual's curiosity about risky behaviors.

From another view, our findings could also increase the desire of the public to understand some aversive information. Curiosity, as an internally driven exploration, could help counteract health information avoidance (Horn et al. 2024). For individuals who deliberately avoid adverse information, we could arouse their relative deprivation sense by previewing attitudes. Thus, we could promote their access to relevant knowledge and information. For example, for elderly people who are unwilling to learn about chronic diseases, we could emphasize the drawbacks of missing information to enhance their willingness.

### 5.3 Limitations and Future Research Directions

This research has some limitations. First, existing research concluded that there is no relationship or only a small effect size between TRB and social approval (Carter et al. 2014; Nicolls et al. 2024). However, we still cannot ignore the impact of social approval on risky behavior (Rulison et al. 2016). Therefore, in the future, it is necessary to explore whether the attitudes of interest curiosity and deprivation curiosity toward TRB are moderated by social approval.

Second, we provided only two options for participants in Study 2: watch and skip. However, in real life, attitudes when confronted with TRBs are complex (Vickers et al. 1990; Zhang et al. 2023). Therefore, future research needs to take this aspect into account as well, for example, by providing a wider variety of attitudinal choices. Additionally, we used only simple TRB materials in this research. The range of traffic behaviors that could cause danger is very wide, especially with the application of smartphones; the dangers associated with mobile phone use while driving cannot be ignored (Vardaki and Yannis 2013). Therefore, future research needs to examine the attitude change of interest curiosity and deprivation curiosity toward other TRB in conformity scenarios.

Finally, in Study 2, the levels of interest curiosity did not show a significant effect on TRB attitude change in the conformity situation, but we could see a weak correlation between interest curiosity and conformity in Study 1. We can explain it by the low need for cognitive closure that characterizes interest curiosity. The conventional view suggests that a high need for cognitive closure enhances individuals' tendency toward conformity (Kruglanski et al. 2006). However, Jia et al. (2014) indicated that individuals with a low need for closure also tend to conform when facing strong norms in the environment. Therefore, in conjunction with Litman's interest/deprivation model of curiosity (Litman 2010), we need to further consider the specification of interest curiosity in the future.

## 6 Conclusion

Our research was based on the uncertainty‐identity theory and interest/deprivation model of curiosity to discuss the difference between the two types of epistemic curiosity. We also explored the explanation of conformity for the relationship between epistemic curiosity and TRB. Results showed that people with deprivation curiosity tend to change their attitudes toward risky behavior to follow the majority after being shown public attitudes, but people with interest curiosity did not. Our findings provided empirical support for the distinction between the two types of epistemic curiosity and exposed the mechanisms behind epistemic curiosity and TRB.

## Ethics Statement

For each study, ethics approval was obtained from the ethics committee of Northwest University. Informed consent was obtained from each participant prior to the experiment.

## Conflicts of Interest

The authors declare no conflicts of interest.

## References

[pchj70024-bib-0001] Anderson, C. L. , D. D. Dixson , M. Monroy , and D. Keltner . 2020. “Are Awe‐Prone People More Curious? The Relationship Between Dispositional Awe, Curiosity, and Academic Outcomes.” Journal of Personality 88, no. 4: 762–779. 10.1111/jopy.12524. 31705660

[pchj70024-bib-0002] Asch, S. E. 1951. “Effects of Group Pressure Upon the Modification and Distortion of Judgments.” In Groups, Leadership and Men; Research in Human Relations, 177–190. Carnegie Press. 10.1525/9780520313514-017.

[pchj70024-bib-0003] Berlyne, D. E. 1954. “A Theory of Human Curiosity.” British Journal of Psychology 45, no. 3: 180–191. 10.1111/j.2044-8295.1954.tb01243.x. 13190171

[pchj70024-bib-0004] Berlyne, D. E. 1962. “Uncertainty and Epistemic Curiosity.” British Journal of Psychology 53, no. 1: 27–34. 10.1111/j.2044-8295.1962.tb00811.x. 13867957

[pchj70024-bib-0005] Carter, P. M. , C. R. Bingham , J. S. Zakrajsek , J. T. Shope , and T. B. Sayer . 2014. “Social Norms and Risk Perception: Predictors of Distracted Driving Behavior Among Novice Adolescent Drivers.” Journal of Adolescent Health 54, no. 5: S32–S41. 10.1016/j.jadohealth.2014.01.008. PMC7189891 24759439

[pchj70024-bib-0006] Comunian, A. L. 1994. “Anger, Curiosity, and Optimism.” Psychological Reports 75, no. 3_suppl: 1523–1528. 10.2466/pr0.1994.75.3f.1523. 7886175

[pchj70024-bib-0007] González‐Iglesias, B. , J. A. Gómez‐Fraguela , and M. A. Luengo‐Martín . 2012. “Driving Anger and Traffic Violations: Gender Differences.” Transportation Research Part F‐Traffic Psychology and Behaviour 15, no. 4: 404–412. 10.1016/j.trf.2012.03.002.

[pchj70024-bib-0008] Hardy, J. H., III , A. M. Ness , and J. Mecca . 2017. “Outside the Box: Epistemic Curiosity as a Predictor of Creative Problem Solving and Creative Performance.” Personality and Individual Differences 104: 230–237. 10.1016/j.paid.2016.08.004.

[pchj70024-bib-0009] Hassan, M. M. , S. Bashir , and P. Mussel . 2015. “Personality, Learning, and the Mediating Role of Epistemic Curiosity: A Case of Continuing Education in Medical Physicians.” Learning and Individual Differences 42: 83–89. 10.1016/j.lindif.2015.07.018.

[pchj70024-bib-0010] Hayes, A. F. 2013. Introduction to Mediation, Moderation, and Conditional Process Analysis: A Regression‐Based Approach. Guilford Press. 10.1111/jedm.12050.

[pchj70024-bib-0011] Hertz, N. , and E. Wiese . 2016. “Influence of Agent Type and Task Ambiguity on Conformity in Social Decision Making.” Proceedings of the Human Factors and Ergonomics Society Annual Meeting 60, no. 1: 313–317. 10.1177/1541931213601071.

[pchj70024-bib-0012] Hess, A. R. B. , and R. M. M. de Almeida . 2019. “Female Crack Cocaine Users Under Treatment at Therapeutic Communities in Southern Brazil: Characteristics, Pattern of Consumption, and Psychiatric Comorbidities.” Trends in Psychiatry and Psychotherapy 41, no. 4: 369–374. 10.1590/2237-6089-2018-0089. 31778427

[pchj70024-bib-0013] Hogg, M. A. 2007. “Uncertainty‐Identity Theory.” Advances in Experimental Social Psychology 39: 69–126. 10.1016/S0065-2601(06)39002-8.

[pchj70024-bib-0014] Hogg, M. A. , J. R. Adelman , and R. D. Blagg . 2010. “Religion in the Face of Uncertainty: An Uncertainty‐Identity Theory Account of Religiousness.” Personality and Social Psychology Review 14, no. 1: 72–83. 10.1177/1088868309349692. 19855094

[pchj70024-bib-0015] Hogg, M. A. , J. T. Siegel , and Z. P. Hohman . 2011. “Groups Can Jeopardize Your Health: Identifying With Unhealthy Groups to Reduce Self‐Uncertainty.” Self and Identity 10, no. 3: 326–335. 10.1080/15298868.2011.558762.

[pchj70024-bib-0016] Horn, S. , Y. Litovsky , and G. Loewenstein . 2024. “Using Curiosity to Counter Health Information Avoidance.” Social Science & Medicine 340: 116383. 10.1016/j.socscimed.2023.116383. 38039766

[pchj70024-bib-0017] Hsee, C. K. , and B. Ruan . 2016. “The Pandora Effect: The Power and Peril of Curiosity.” Psychological Science 27, no. 5: 659–666. 10.1177/0956797616631733. 27000178

[pchj70024-bib-0018] Huang, Y. , K. M. Kendrick , and R. Yu . 2014. “Conformity to the Opinions of Other People Lasts for No More Than 3 Days.” Psychological Science 25, no. 7: 1388–1393. 10.1177/0956797614532104. 24855020

[pchj70024-bib-0019] Huck, J. T. , E. A. Day , L. Lin , A. G. Jorgensen , J. Westlin , and J. H. Hardy . 2020. “The Role of Epistemic Curiosity in Game‐Based Learning: Distinguishing Skill Acquisition From Adaptation.” Simulation & Gaming 51, no. 2: 141–166. 10.1177/1046878119895557.

[pchj70024-bib-0020] Jia, L. , E. R. Hirt , and D. N. Evans . 2014. “Putting the Freeze on Priming: The Role of Need for Cognitive Closure on the Prime‐Norm Dynamic.” Personality and Social Psychology Bulletin 40, no. 7: 931–942. 10.1177/0146167214530435. 24769737

[pchj70024-bib-0021] Jongenelis, M. I. , E. Brennan , T. Slevin , et al. 2019. “Factors Associated With Intentions to Use e‐Cigarettes Among Australian Young Adult Non‐Smokers.” Drug and Alcohol Review 38, no. 5: 579–587. 10.1111/dar.12963. 31317596

[pchj70024-bib-0022] Jovanovic, V. , and D. Brdaric . 2012. “Did Curiosity Kill the Cat? Evidence From Subjective Well‐Being in Adolescents.” Personality and Individual Differences 52, no. 3: 380–384. 10.1016/j.paid.2011.10.043.

[pchj70024-bib-0023] Kang, M. J. , M. Hsu , I. M. Krajbich , et al. 2009. “The Wick in the Candle of Learning: Epistemic Curiosity Activates Reward Circuitry and Enhances Memory.” Psychological Science 20, no. 8: 963–973. 10.1111/j.1467-9280.2009.02402.x. 19619181

[pchj70024-bib-0024] Kochetova, T. V. 2022. “The Patterns of Drivers' Traffic Behavior: Evidence From Three Countries.” Frontiers in Psychology 13: 869029. 10.3389/fpsyg.2022.869029. 35465507 PMC9021888

[pchj70024-bib-0025] Kruglanski, A. W. , A. Pierro , L. Mannetti , and E. De Grada . 2006. “Groups as Epistemic Providers: Need for Closure and the Unfolding of Group‐Centrism.” Psychological Review 113, no. 1: 84–100. 10.1037/0033-295X.113.1.84. 16478302

[pchj70024-bib-0026] Kruglanski, A. W. , and D. M. Webster . 1991. “Group Members' Reactions to Opinion Deviates and Conformists at Varying Degrees of Proximity to Decision Deadline and of Environmental Noise.” Journal of Personality and Social Psychology 61, no. 2: 212–225. 10.1037/0022-3514.61.2.212. 1920063

[pchj70024-bib-0027] Larsen, K. S. 1974. “Conformity in the Asch Experiment.” Journal of Social Psychology 94, no. 2: 303–304. 10.1080/00224545.1974.9923224.

[pchj70024-bib-0028] Lauriola, M. , J. A. Litman , P. Mussel , R. De Santis , H. M. Crowson , and R. R. Hoffman . 2015. “Epistemic Curiosity and Self‐Regulation.” Personality and Individual Differences 83: 202–207. 10.1016/j.paid.2015.04.017.

[pchj70024-bib-0029] Lemarié, L. , F. Bellavance , and J. C. Chebat . 2019. “Regulatory Focus, Time Perspective, Locus of Control and Sensation Seeking as Predictors of Risky Driving Behaviors.” Accident Analysis & Prevention 127: 19–27. 10.1016/j.aap.2019.02.025. 30826693

[pchj70024-bib-0030] Lennox, R. D. , and R. N. Wolfe . 1984. “Revision of the Self‐Monitoring Scale.” Journal of Personality and Social Psychology 46, no. 6: 1349–1364. 10.1037/0022-3514.46.6.1349. 6737217

[pchj70024-bib-0031] Li, T. , H. Y. Huang , J. Liu , and X. Tang . 2023. “Killing the Cats or Satisfying the Human? The Role of Epistemic Curiosity in Adolescents' Multidimensional Well‐Being.” Journal of Pacific Rim Psychology 17: 1–10. 10.1177/18344909231185381.

[pchj70024-bib-0032] Litman, J. A. 2008. “Interest and Deprivation Factors of Epistemic Curiosity.” Personality and Individual Differences 44, no. 7: 1585–1595. 10.1016/j.paid.2008.01.014.

[pchj70024-bib-0033] Litman, J. A. 2010. “Relationships Between Measures of I‐ and D‐Type Curiosity, Ambiguity Tolerance, and Need for Closure: An Initial Test of the Wanting‐Liking Model of Information‐Seeking.” Personality and Individual Differences 48, no. 4: 397–402. 10.1016/j.paid.2009.11.005.

[pchj70024-bib-0034] Litman, J. A. , and T. L. Jimerson . 2004. “The Measurement of Curiosity as a Feeling of Deprivation.” Journal of Personality Assessment 82, no. 2: 147–157. 10.1207/s15327752jpa8202_3. 15041521

[pchj70024-bib-0035] Litman, J. A. , and C. D. Spielberger . 2003. “Measuring Epistemic Curiosity and Its Diversive and Specific Components.” Journal of Personality Assessment 80, no. 1: 75–86. 10.1207/S15327752JPA8001_16. 12584070

[pchj70024-bib-0036] Loewenstein, G. 1994. “The Psychology of Curiosity: A Review and Reinterpretation.” Psychological Bulletin 116, no. 1: 75–98. 10.1037/0033-2909.116.1.75.

[pchj70024-bib-0037] Ma, J. 2023. “Curious Supervisor Puts Team Innovation Within Reach: Investigating Supervisor Trait Curiosity as a Catalyst for Collective Actions.” Organizational Behavior and Human Decision Processes 175: 104236. 10.1016/j.obhdp.2023.104236.

[pchj70024-bib-0038] Mechera‐Ostrovsky, T. , S. X. Liew , and B. R. Newell . 2023. “The Role of Risk, Regret, and Rejoice in Non‐Instrumental Information Seeking.” Journal of Behavioral Decision Making 36, no. 1: e2294. 10.1002/bdm.2294.

[pchj70024-bib-0039] Mo, P. K. H. , and W. S. M. Winnie . 2010. “The Influence of Health Promoting Practices on the Quality of Life of Community Adults in Hong Kong.” Social Indicators Research 95: 503–517. 10.1007/s11205-009-9523-9.

[pchj70024-bib-0040] Nicolls, M. , V. Truelove , and K. B. Stefanidis . 2024. “How Do Perceptions of Others' Approval of, and Engagement in, Hand‐Held Phone Use Influence Young Drivers? A Mixed‐Method Study.” Safety Science 176: 106546. 10.1016/j.ssci.2024.106546.

[pchj70024-bib-0041] Niehoff, E. , and S. Oosterwijk . 2020. “To Know, to Feel, to Share? Exploring the Motives That Drive Curiosity for Negative Content.” Current Opinion in Behavioral Sciences 35: 56–61. 10.1016/j.cobeha.2020.07.012.

[pchj70024-bib-0042] Oosterwijk, S. 2017. “Choosing the Negative: A Behavioral Demonstration of Morbid Curiosity.” PLoS One 12, no. 7: e0178399. 10.1371/journal.pone.0178399. 28683147 PMC5500011

[pchj70024-bib-0043] Ouyang, Y. , K. M. Kincaid , D. E. Rast , A. M. Gaffney , and M. A. Hogg . 2024. “Incumbency and Self‐Uncertainty: When Prototypical Leaders Lose Their Advantage.” Journal of Social Psychology 165, no. 2: 189–206. 10.1080/00224545.2024.2325420. 38452797

[pchj70024-bib-0044] Pierce, J. P. , J. M. Distefan , R. M. Kaplan , and E. A. Gilpin . 2005. “The Role of Curiosity in Smoking Initiation.” Addictive Behaviors 30, no. 4: 685–696. 10.1016/j.addbeh.2004.08.014. 15833574

[pchj70024-bib-0045] Qian, Y. N. , Q. N. Sun , G. Q. Fei , et al. 2020. “Riding Behavior and Electric Bike Traffic Crashes: A Chinese Case‐Control Study.” Traffic Injury Prevention 21, no. 1: 24–28. 10.1080/15389588.2019.1696963. 31846600

[pchj70024-bib-0046] Rosenbloom, T. , Y. Sapir‐Lavid , and A. Perlman . 2016. “Risk Factors in Road Crossing Among Elderly Pedestrians and Readiness to Adopt Safe Behavior in Socio‐Economic Comparison.” Accident Analysis & Prevention 93: 23–31. 10.1016/j.aap.2016.04.004. 27155211

[pchj70024-bib-0047] Rulison, K. L. , E. Wahesh , D. L. Wyrick , and W. DeJong . 2016. “Parental Influence on Drinking Behaviors at the Transition to College: The Mediating Role of Perceived Friends' Approval of High‐Risk Drinking.” Journal of Studies on Alcohol and Drugs 77, no. 4: 638–648. 10.15288/jsad.2016.77.638. 27340969

[pchj70024-bib-0048] Ryb, G. E. , P. C. Dischinger , J. A. Kufera , and K. M. Read . 2006. “Risk Perception and Impulsivity: Association With Risky Behaviors and Substance Abuse Disorders.” Accident Analysis & Prevention 38, no. 3: 567–573. 10.1016/j.aap.2005.12.001. 16426559

[pchj70024-bib-0049] Sekiguchi, T. 2023. “Curiosity Makes Your Mind Wander: Effects of Epistemic Curiosity and Trait Anxiety on Mind Wandering.” Personality and Individual Differences 204: 112069. 10.1016/j.paid.2022.112069.

[pchj70024-bib-0050] Shen, M. , P. Liu , X. Li , J. Zhou , and H. Chen . 2020. “The Gilding‐The‐Lily Effect: Exploratory Behavior Energized by Curiosity.” Frontiers in Psychology 11: 1381. 10.3389/fpsyg.2020.01381. 32719635 PMC7350549

[pchj70024-bib-0051] Smith, J. R. , M. A. Hogg , R. Martin , and D. J. Terry . 2007. “Uncertainty and the Influence of Group Norms in the Attitude‐Behaviour Relationship.” British Journal of Social Psychology 46, no. 4: 769–792. 10.1348/014466606X164439. 18062848

[pchj70024-bib-0052] Swan, G. E. , and D. Carmelli . 1996. “Curiosity and Mortality in Aging Adults a 5‐Year Follow‐Up of the Western Collaborative Group Study.” Psychology and Aging 11, no. 3: 449–453. 10.1037/0882-7974.11.3.449. 8893314

[pchj70024-bib-0053] Tang, T. , Y. Guo , X. Zhou , S. Labi , and S. Zhu . 2021. “Understanding Electric Bike Riders' Intention to Violate Traffic Rules and Accident Proneness in China.” Travel Behaviour and Society 23: 25–38. 10.1016/j.tbs.2020.10.010.

[pchj70024-bib-0054] Tang, X. , and K. Salmela‐Aro . 2021. “The Prospective Role of Epistemic Curiosity in National Standardized Test Performance.” Learning and Individual Differences 88: 102008. 10.1016/j.lindif.2021.102008.

[pchj70024-bib-0055] Ulleberg, P. , and T. Rundmo . 2003. “Personality, Attitudes and Risk Perception as Predictors of Risky Driving Behaviour Among Young Drivers.” Safety Science 41, no. 5: 427–443. 10.1016/S0925-7535(01)00077-7.

[pchj70024-bib-0056] Vardaki, S. , and G. Yannis . 2013. “Investigating the Self‐Reported Behavior of Drivers and Their Attitudes to Traffic Violations.” Journal of Safety Research 46: 1–11. 10.1016/j.jsr.2013.03.001. 23932680

[pchj70024-bib-0057] Vickers, R. R., Jr. , T. L. Conway , and L. K. Hervig . 1990. “Demonstration of Replicable Dimensions of Health Behaviors.” Preventive Medicine 19, no. 4: 377–401. 10.1016/0091-7435(90)90037-K. 2399221

[pchj70024-bib-0058] Whitecross, W. M. , and M. Smithson . 2023a. “Curiously Different: Interest‐Curiosity and Deprivation‐Curiosity May Have Distinct Benefits and Drawbacks.” Personality and Individual Differences 213: 112310. 10.1016/j.paid.2023.112310.

[pchj70024-bib-0059] Whitecross, W. M. , and M. Smithson . 2023b. “Open or Opposed to Unknowns: How Do Curious People Think and Feel About Uncertainty?” Personality and Individual Differences 209: 112210. 10.1016/j.paid.2023.112210.

[pchj70024-bib-0060] Wu, B.‐P. , and L. Chang . 2012. “The Social Impact of Pathogen Threat: How Disease Salience Influences Conformity.” Personality and Individual Differences 53, no. 1: 50–54. 10.1016/j.paid.2012.02.023.

[pchj70024-bib-0061] Yang, G. , L. Kexin , and L. Hong . 2021. “Go With the Flow Against Uncertainty About Self Under Existential Threat.” Self and Identity 20, no. 3: 438–461. 10.1080/15298868.2020.1737569.

[pchj70024-bib-0062] Zedelius, C. M. , M. E. Gross , and J. W. Schooler . 2022. “Inquisitive but Not Discerning: Deprivation Curiosity Is Associated With Excessive Openness to Inaccurate Information.” Journal of Research in Personality 98: 104227. 10.1016/j.jrp.2022.104227.

[pchj70024-bib-0063] Zhang, W. , H. Guo , C. Wang , et al. 2023. “Analysis of Pedestrian Illegal Crossing at Unmarked Segments: Environmental Factors, Pedestrian Characteristics and Crossing Behaviours.” Transportation Research Part F: Traffic Psychology and Behaviour 99: 339–355. 10.1016/j.trf.2023.10.022.

[pchj70024-bib-0064] Zhou, R. G. , and W. J. Horrey . 2010. “Predicting Adolescent Pedestrians' Behavioral Intentions to Follow the Masses in Risky Crossing Situations.” Transportation Research Part F: Traffic Psychology and Behaviour 13, no. 3: 153–163. 10.1016/j.trf.2009.12.001.

[pchj70024-bib-0065] Zhou, R. G. , W. J. Horrey , and R. F. Yu . 2009. “The Effect of Conformity Tendency on Pedestrians' Road‐Crossing Intentions in China: An Application of the Theory of Planned Behavior.” Accident Analysis and Prevention 41, no. 3: 491–497. 10.1016/j.aap.2009.01.007. 19393798

